# Living things are showing increasing anomalies in their seasonal activity, which could disrupt the dynamics of biodiversity and ecosystems

**DOI:** 10.1038/s41598-025-16585-2

**Published:** 2025-09-25

**Authors:** Isabelle Chuine, Iñaki Garcia de Cortazar-Atauri, Frédéric Jean, Colin Van Reeth

**Affiliations:** 1 CEFE, Univ Montpellier, CNRS, EPHE, IRD, Montpellier, France; 2 INRAE, US AgroClim, Avignon, 84914 France; 3 INRAE, URFM, Avignon, 84914 France; 4 CREA Mont Blanc, Chamonix, France

**Keywords:** Phenology, Abnormal activity, Citizen science, Climate change, Ecology, Climate sciences, Ecology, Environmental sciences, Environmental social sciences

## Abstract

The IPCC reported that the 2011–2020 decade has been the warmest on record worldwide. The frequency of climate extremes has increased and the future rates of warming will be well above historical averages. Climate warming has already modified species seasonal activity with cascading effects on species interactions, geographical ranges, ecosystems productivity and feedback to the atmosphere. However, we lack information on how the new climate regime will translate in terms of impacts for human populations and ecosystems. Here we report on abnormal seasonal activities of plants and animals, which took place in Europe and other countries worldwide since 2015. We show that they are unpreceded, related to warmer fall and winter as well as drier summer conditions. These anomalies are projected to increase in frequency in a near future and might have dramatic consequences for biodiversity dynamics, ecosystems functioning and human activities.

**Supplementary Information:**

The online version contains supplementary material available at 10.1038/s41598-025-16585-2.

## Introduction

Global climate models had forecasted that 2020 would mark the beginning of an acceleration of global warming worldwide (IPCC, 2013). The Intergovernmental Panel on Climate Change reported that the last decade (2011–2020) had been the warmest on record worldwide, approximatively 1.09 °C above pre-industrial values, with the 2015–2021 period being the warmest on record^2,3^. The World Meteorological Organization reported that the last nine years (2015–2023) have been the warmest on record despite an unusual three-year lasting La Niña event^4^ and 2024 might be added to this series of records. We are currently in a transition period to a climate regime that will be dominated by increasing greenhouse gas forcing with background rates of warming well above historical averages^5^.

Impacts of global climate change on human health, water, food, economy, infrastructure, security, biodiversity, and nature’s contributions to people are reported worldwide constantly, becoming a broad threat to humanity^6^. Along with this change in climate trend, we are observing a significant increase in the frequency of extreme climatic events^3^. Unusual meteorological events are usually highly localized and have unpreceded effects on ecosystems, human populations and their activities^7–9^. For this reason, although we know we are indeed moving towards a change in climate regime^10^ we still lack reports on how the increase in the rate of climate change will translate specifically in terms of impacts. Although climatic trends have shown some break points in the past, such as in the 1980s^11^ they did not lead to major disruption of the biology of living beings so far. However, an increasing number of articles highlight a rising concern among scientists, decision makers and the large public about irreversible changes^3,12,13^.

Primary adaptation of species to changing environmental conditions relies in their capacity to modulate their physiological activity seasonally, and noticeably to cease or reduce it drastically during unfavorable environmental conditions. In extratropical regions, the annual cycle of plants is usually characterized by the occurrence of single bud flush, flowering, and fruit production because the temperature conditions do not allow for multiple ones. Each plant species, whatever wild or cultivated, shows a precise timing of its developmental cycle, which has enabled it to survive and reproduce successfully in particular climatic conditions called the climatic niche of the species^14^. Until recently, we had no precise data on the timing of occurrence of the different steps of the annual cycle of species, excepting some crops because this timing is crucial for the success of the harvest^15,16^ (see also FAO Crop calendar). Thanks to citizen science programs (e.g. USA-NPN, Nature’s Calendar, Observatoire Des Saisons, Phenoclim) and long-term observation networks (e.g. PEP725, TEMPO) which have been running since the late nineteen century for some of them, we have now a precise overview of the timing of the annual cycle of many of plants species in different climatic regions, sometimes far prior 1950.

These observations have shown that climate change has modified the timing of the annual cycles of a vast number of plant and animal species and have allowed quantifying precisely those changes in different geographical regions^17,18^. Changes have affected food webs whatever the trophic level by desynchronizing the annual cycles of species interacting in these webs^19–21^ as well as the yield of many crops^22,23^ and feedbacks of the surface to the atmosphere^24^. Changes correspond so far to an advancement of the onset of spring activity and a delay of activity cessation at fall^17,18^. While trends of earlier growth onset have been linear so far, some studies have shown that they had weakened after 2000 in plants^25–27^ and that this is because plant cell activity does not respond linearly to temperature^28,29^. Indeed, all studies point out that outside the tropics, plants need cold during winter to develop subsequently normally at spring. Warming of winter can therefore be detrimental to plant development because it might counteract the positive effect of warming at spring which accelerates development.

Here we report on unpreceded seasonal activities in wildlife and crops that have been occurring since 2015 worldwide, and will have dramatic consequences on agroecosystem productivity, nature contributions to people, and biodiversity dynamics if they become more frequent. We first report on multiple occurrences within a year of normally unique biological events in Western Europe, Northern America and other countries. Second, we analyze the particular winter 2015–2016 which showed massive abnormal phenological events. We compare the seasonal activity of 22 species during that winter to their activity since 1900, and we point to the meteorological conditions that gave rise to these anomalies and how they might have affected the physiology of the species. Third, we reveal using historical documents that the anomalies reported since 2015 in species seasonal activity are unpreceded over the last millennium. Finally, we discuss the societal and economic consequences of such events, as well as the scientific questions that arise from these observations, and recommendations to tackle them.

## Results

### Wildlife and crops show abnormal seasonal activities since 2015

**Fig. 1:**
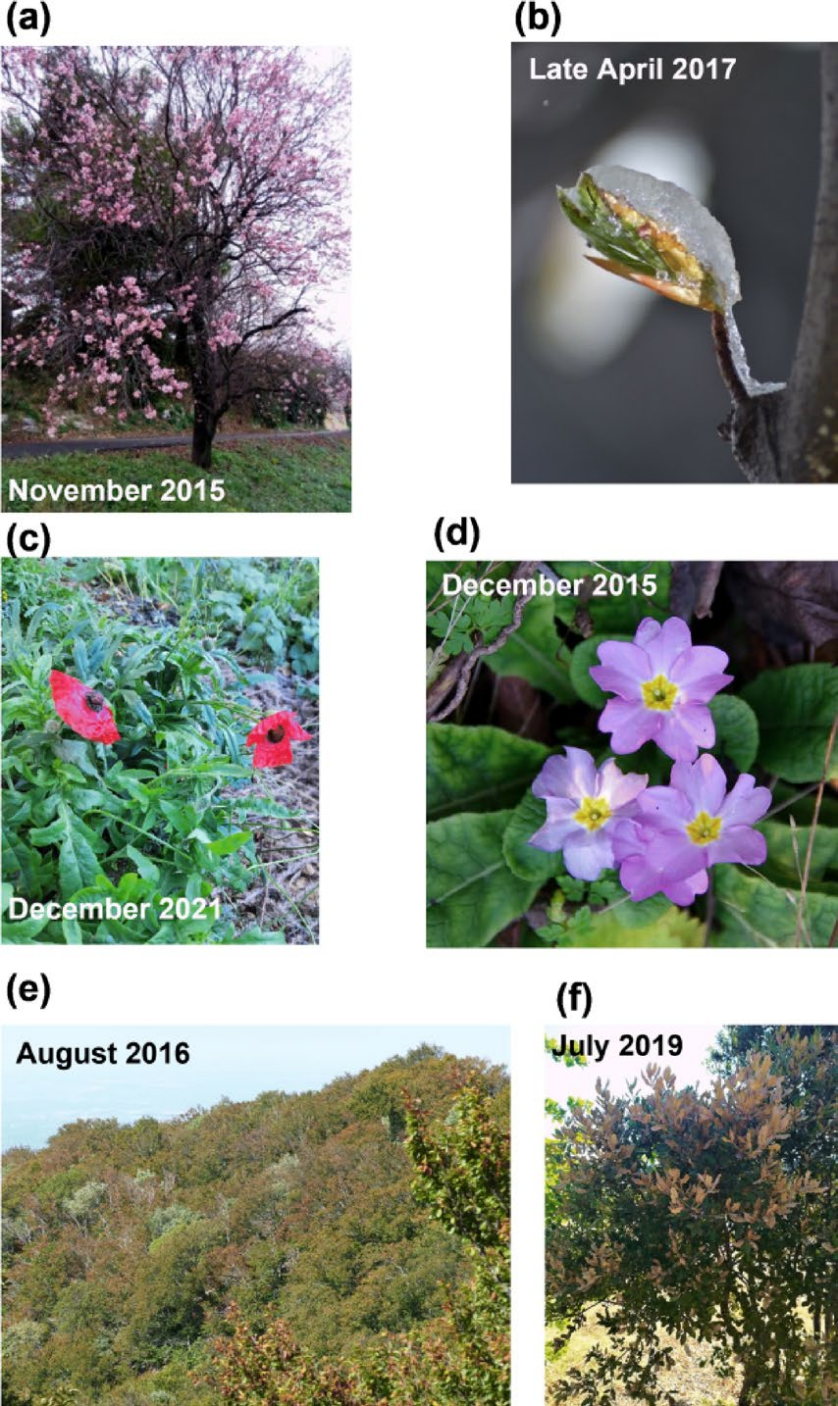
Photographs of examples of phenological anomalies: (**a**) *Prunus* in full bloom in October in France; (**b**) beech flushing at 1200 m in the French Alps during a massive frost event that subsequently killed the leaves on May 1st 2017; (**c**) poppy in bloom in December in Luxembourg; (**d**) primerose blooming in October in northern France; (**e**) beech trees with leaves already senescent in August in southern France (Mont Ventoux); (f). Holm oak (Mediterranean species) leaves burned by the European heat wave of June 2019 in southern France.

Abnormal seasonal activities have been observed repeatedly since 2015 in extratropical regions, and especially in Western Europe and North America where citizen science programs are the most active (Table 1; Fig. 1). There have also been reported in the traditional media in these areas as well as Asia, Australia, and South America. A special feature of the data reporting such events is that they are not listed in most conventional databases for several reasons. Such observations have been reported, for most of them, by people participating to citizen-science programs, or active in social media, and by journalists in traditional media (Table S1).

**Table 1:** Description of the abnormal seasonal activities reported since 2015 in extratropical regions, the Climatic conditions responsible for such responses with their forecasted frequency after 2020, the biological response leading to this type of events, and the consequences of such events. See table S1a, S1b for related data and Fig. 1 for pictures.

Event	Climatic conditions	Frequency post 2020	Biological response	Consequences
Late summer, autumn and winter leaf flush and flowering (Fig. 1a-d)	Increased temperature in autumn	Increased	No dormancy induction at autumn	Frost damage (Fig. 1b) Leaf malformation Mismatch with pollinators Fruit abortion Resources loss Weakening of the plant
	Increased temperature in winter following a short cold spell in autumn	Increased	Very early dormancy break	
	Heat wave, Drought, Storm	Increased	Dormancy break following a destruction of the foliage	
Erratic and long lasting flowering	Increased winter temperature	Increased	Incomplete dormancy break	Flower malformation Increased mismatch between trees – decreased pollination success and yield
Very late leaf senescence	Increased temperature in autumn without pronounced summer drought summer	Decreased at higher latitudes Increased at lower latitudes	Absence of cues triggering leaf senescence	Increased growing season length that could yield higher carbon storage
Very early leaf senescence (Fig. 1e)	Increased summer drought	Increased	Early cues of leaf senescence	Decreased growing season length that could yield lower carbon storage

The vast majority of the reports concern flowering and leaf flush in autumn and winter of plants normally flushing in spring.

Most of these situations have been related to above normal temperature conditions in autumn and winter, and drier and warmer conditions in summer. This season shift in leaf flush and blooming results most of the time in a destruction of the leaves and flowers by subsequent frost events during winter. Devastating frost damages due to off-season bud flush have been particularly frequent since 2015 in orchards and vineyards of Western Europe, damaging mostly fruit trees and grapevine but also forest trees and crops (Fig. 1b). Such damages have been particularly important in springs 2016, 2020 and 2021, leading to very large economic loss^30,31^. The second most frequent abnormal event reported concerns mostly fruit trees so far, and is an erratic and spread out flowering due to insufficient cold during winter that prevent a complete release of flower buds dormancy. This resulted in particular to low fertilization success and decreased fruit yield^32,33^. Other events reported are either very late or very early leaf color change following respectively high autumn temperature (without summer heat wave and drought) and summer drought. Some animal species also showed unpreceded seasonal activity change, such as sea breams, which did not migrate southward and remained in their spawning sites, amphibians which showed very much spread-out migration dates due the absence of the cues associated to normal winter conditions, or dragonfly emerging in mi-autumn (Table S2).

### The exceptional winter 2015–2016 in Western Europe

**Fig. 2:**
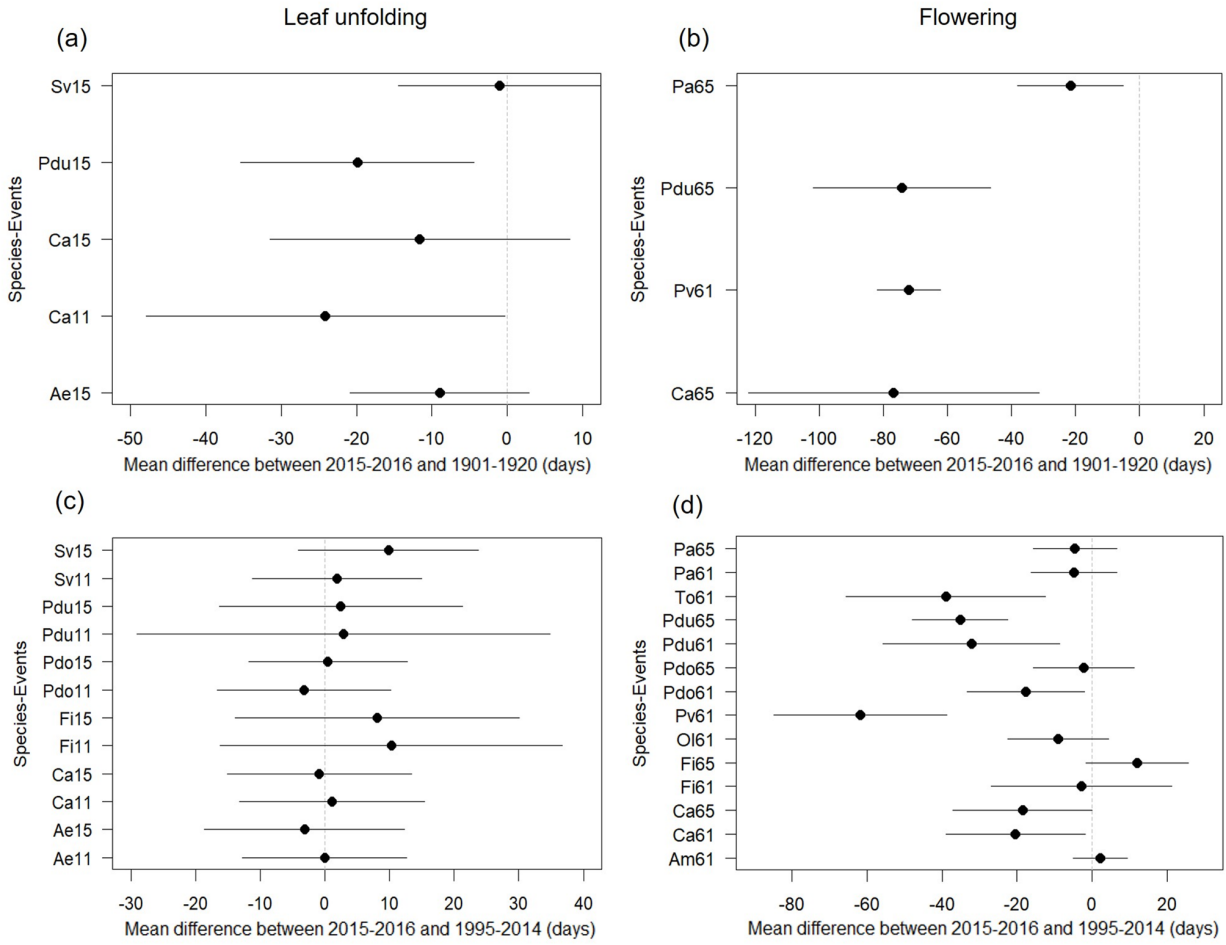
Difference (mean and standard deviation) in the date of occurrence of leaf unfolding (**a**,** c**) and flowering (**b**,** d**) between the winter-spring 2015–2016 and 1901–1920 (first row) periods and 1995–2014 (second row) in France. Ae *Aesculus hippocastanum*, Am *Anacamptis morio*, Ca *Corylus avellana*, Fi *Forsythia intermedia*, Ol *Ophrys lupercalis*, Pa *Prunus avium*, Pdo *Prunus domestica*, Pdu *Prunus dulcis*, Sv *Syringa vulgaris*, To *Taraxacum officinale*. 11: BBCH 11, 10% of the leaves are unfold; 15: BBCH 15, 50% of the leaves are unfold; 61: BBCH 61, 10% of the flowers are mature; 65: BBCH 65, 50% of the flowers are mature. BBCH refers to the international scale to describe plant annual cycle^34^. Data are from Table S3.

Among the abnormal events that we report above, those which occurred during the winter 2015–2016, were the most striking as regard to their nature, span and amplitude. Flowering was the most affected event and was earlier by up to 80 days compared to 1901–1920 when climate change was not detectable yet, and 40 days compared to the warmest period of 1995–2014 (Fig. 2, see Material & Methods for the choice of the time periods compared). This resulted in a season shift of flowering from spring to autumn or winter in four of the ten species for which the analysis could be carried out. In addition to the massive flowering change in these four species, many other species also showed signs of season change in their flowering that had never been recorded before (Table S2). Most of these species are ornamental herbaceous species, ornamental trees and fruit trees. Comparatively, leaf unfolding showed no massive shift relative to the warmest period 1995–2014, but large shift relative to the colder period 1901–1920, with much larger variation among observations (Fig. 2). In addition, some tree species such as poplars and Judean tree did not shed their leaves, which remained green all winter, falling only in spring and causing a delayed bud break (Table S2). Temperature data of the 2015–2016 winter show that September-October temperatures were lower than normal (reference period 1961–1990) by 0.77 °C while those of November, December, January and February were warmer than normal by 2.69 °C (Fig. S1).

### Abnormal seasonal events of the 2010s are unpreceded

The first question that arises regarding these observations is whether they are as exceptional as they seem to be. In Western Europe, several years have been identified as exceptional in terms of plant seasonal activity during the last millennium (Table 2). All these unusual observations occurred during exceptionally warm autumns or winters, and for some of them warm and dry summers, and two of them took place at the end of the Medieval Climate Optimum (c. 950 to c. 1250). During the second part of the last millennium, all extreme climatic situations reported by climate historians concern cold situations^35,36^ and none of them were related to abnormal phenological events as those we report here for the 2010s.

**Table 2:** Previous abnormal phenological events reported in Europe since the 12th century in chronicles.

Years	Species	Phenological anomaly	References
1116–1117	Strawberries	Flowering in autumn and fruit ripe at Christmas	^37^
1186–1187	Fruit trees Birds	Trees flowering and birds nesting in December-January	^37^
1289–1290	Trees Grapevine	No leaf color change in autumn Flowering in January	^37,38^
1327–1328	Fruit trees Grapevine	Flowering in January Flowering in April, ripe late July	^38^
1420	Fruit trees Grapevine	Bud break by March 20th Flowering in April, ripe early August	^38^
1426	Trees	Second bud flush in November	^38^
1858	Fruit trees, shrubs, herbaceous	Second autumn flowering from mid-October to early November	^39^
1921	Fruit trees, ornamental shrubs, herbaceous	Second autumn flowering in October	^40^

In the recent years, a similar event has been observed during the winter 2006–2007 in Germany where hazel and snowdrop flowering have been reported to occur on average 37 and 22 days earlier respectively compared to the preceding 56 years; and earlier by 31.7 and 15.4 days compared to the preceding 25 years^36^. Similar observations have been reported for orchids in southern France as well, with 3 weeks advance compared to the previous earliest record of 2003 ^41^. The winter 2015–2016 went a step further in the extreme. This abnormal situation might be related to the fact that temperatures of the early autumn (September, October) were much lower (mean anomaly − 0.77 °C) than in 2006 (mean anomaly + 2.70 °C), while the rest of the autumn and winter (November to February) was much warmer (mean anomaly + 2.69 °C) than the winter 2006–2007 (mean anomaly + 1.94 °C) (Fig. S1). To conclude, abnormal phenological events, especially second blooms in autumn, had occurred in the past thousand years but they represented isolated events decades and hundreds of years apart. The lack of precise data does not allow us to evaluate whether some of these events represented stronger season shift than the ones since 2015 we report here. However, the data we analyzed show that these later are unprecedented and have been more frequent at least since 1901.

## Discussion

The abnormal phenological events observed repeatedly since 2015 bring several important questions. A first set of questions relates to the major consequences that abnormal phenological events have on species survival, species interactions, ecosystems functioning, human societies, and the economy. The risk of massive blooms in the fall could endanger the production of certain perennial fruit crops. Indeed, fall bloom cannot lead to a complete development of the fruits and a second yield because of both low fertilization rate due to low activity of pollinators at this period, and nonoptimal light and temperature conditions to accumulate photosynthesis products in the fruits. This calls for identifying varieties the most susceptible to bloom at fall and maybe anticipate changing varieties in certain regions. Note that desynchronization between the activity of pollinators and fruit trees blooming could also severely affect honey production. Autumn bloom and leaf flush might also deeply modify the seasonal dynamics of resource allocation, jeopardizing individuals’ survival. Indeed, before global warming reaches a level when frost never occurs anymore and winter conditions are warm enough to allow photosynthesis and growth, newly formed organs in autumn are destroyed by autumn and winter frosts (Fig. 1b), and do not play their role of resource acquisition for leaves and seed production for flowers. This results additionally in an important net resource loss for the plant, which has a deleterious effect, potentially leading to death. Perennial species (e.g. forest trees, shrubs, fruits trees) are among the most affected species, with phenological shifts leading in the short term to frost damages, fruit development failure and no yield, and on the long-term plants weakening, putting them at higher risk of pathogen and herbivore attacks. This last issue might become more acute in the future since pathogens and herbivores are able to adapt rapidly their seasonal activity to new climatic conditions^42^. Although reports of abnormal activity in animals were a small minority in the data we present here (Tables S1, S2), documented examples of winter emergence of amphibians and insects with consequences on reproductive success and population density are increasing^43^. Season shifts in activity also disrupt species interactions and food webs. For examples, fruit trees blooming in winter are usually not pollinated because pollinators are not active at this period; and adult forms of insects and amphibians emerging in winter do not find the preys they usually feed on. Finally, season shift in plant and animal activity could also affect human health issues and the way they are handled such as allergy to pollen, tourism economy strongly related to the phenology of emblematic species such as cherry trees in Japan or deciduous forest trees in Canada, and urban ornamental vegetation management.

A second set of questions concern the gap in our understanding of how environmental conditions regulate living beings’ activity. If the occurrence of abnormal activity can help improving our understanding of the regulation of plant and animal seasonal activities, we are, so far, unable to explain how regulation pathways generate them. Based on our current knowledge, shifts of leaf flush and blooming in autumn and winter could be explained by two different situations depending on the climatic context. First, dormancy might not be induced in the buds in early autumn due to abnormally high temperature, so that they continue their development and flush in autumn or early winter. This situation however necessitates that tree lost most of the leaves subsequently to an abnormally hot and dry summer which would suppress the paradormancy induced by these active sinks on newly formed buds. Second, endodormancy could start to settle in early autumn but be quickly broken by a short cold spell, and subsequent warmer than normal autumn-winter conditions could then trigger bud cell growth, leading to bud break in autumn or winter. The low temperature conditions in September-October followed by the abnormally high temperature conditions from November to February during the winter 2015–2016 in Western Europe corresponds to this latter situation. However, if the two situations seem possible, none of them have been proven to be responsible for what happened the winters 2015–2016 and 2006–2007.

A third set of questions relates to our current ability to forecast such abnormal activities and their consequences in a near future. Despite obviously incomplete, our understanding of how environmental conditions regulate plant development has been used to develop phenology models during the last 50 years for crops and forest trees^44^. From now on, these models forecast that the advancement of growth onset that has been observed the last 60 years will progressively decrease in the next decades, and might occur later than it used to by the end of the 21th century because of winter warming delaying bud dormancy break. At warmer species range limits, winter temperature conditions might even become too warm by the end of the 21th century so that dormancy might never be released^28,45^. Other models simulating fruit growth and development predict decreased yield and altered fruit and seed quality^46^. Although economic and ecological consequences of these forecasted impacts are very important, none of the current models is able to predict the anomalies we report here although they have been efficient to predict species seasonal activity so far. In other words, current models are not transferable to certain climatic conditions we have already experienced that might become more frequent in the future because the effects of these conditions have never been investigated and are not represented in the model. Although transferability is a well-known problem in forecasting science especially in natural systems^47,48^ we believe that in the case of process-based phenology models, there is space for model improvement, provided that experimental work allowing to investigate the effects of abnormally warm and dry summers and abnormally warm falls on bud development can be carried out.

A last set of questions concern the way data are collected and subsequently analyzed. Most scientific monitoring programs are currently unable to collect information on abnormal phenological events because their protocols are not adapted. The occurrence of extreme phenological events also prevent the use of classical statistical technics for detecting potentially erroneous data which are usually based on data distribution, affecting data quality check processes. We urgently need to develop new statistical technics and methods of data quality check to separate erroneous data from truly abnormal data.

It is still uncertain whether the abnormal seasonal activities we report in this study will become more frequent in the future or if they are the result of a transitory situation. However, the number of questions they raise and the importance of their consequences for biodiversity and ecosystems call for a special attention to their monitoring, analysis and understanding.

## Methods

### Data

#### Phenology reports in media

A review of reports of abnormal phenological events in traditional media through Google and the social media Twitter since 2015 was realized using the following keywords: in English: strange flowering/blooming, early flowering, in French: floraison précoce/atypique, in Spanish: floracion precoz/atipica. Results of this review are presented in Table S1.

#### Abnormal phenology reports from citizen science programs

We collected abnormal phenology reports realized on plants and animals throughout France since autumn 2015 by the citizen science programs Observatoire des Saisons (https://obs-saisons.fr), Phenoclim (https://phenoclim.org/), Abiome (https://abiome.assoconnect.com/page/1005353-accueil), Orchisauvage (https://www.orchisauvage.fr/) and by volunteers of the TEMPO Network (https://tempo.pheno.fr). This represented a total of 97 data presented in Table S2, and 1121 data for 22 plant species presented in Table S3 for which historical data existed and could be compared to.

#### Historical phenology date

The TEMPO portal (https://data.pheno.fr) provides phenological data for France. More than 2.5. 10^6^ data are currently available for 2044 taxa, 12,225 locations, from 1349 (for the oldest records) to present. A data corresponds to a date of a particular phenological event for a particular species at one location one year. From this portal, we extracted 9842 dates from 1901 to 2014 of phenological events concerning the 22 plant species for which abnormal phenological events had been reported by citizen programs. These data are presented in Table S3.

#### Historical documents

We consulted several articles and documents reporting on past abnormal phenological and climatic events. These documents are presented in Table 2.

### Analysis

From data presented in Table S3, we first determined the amount of data available for the 2015–2016 autumn to spring and for two earlier 20-year periods for the 22 species. The two earlier 20-year periods chosen were 1901–1920 and 1995–2014 based on their distance to 2015–2016 and the amount of data available. We kept for the analysis the data from a species x phenological event when above 15 for the 2015–2016 autumn to spring period and above 50 for the 20-year earlier periods with an average of 270 and 300 data for 1995–2014 and 1901–1920 respectively. This generated a subset of data for leaf unfolding date and flowering date for 11 species that could be compared between the three time periods. For these 11 species, we calculated for each species and each phenological event the differences between the mean date of occurrence during the winter-spring 2015–2016 and the mean date of occurrence over the 1995–2014 period on one hand and over the 1901–1920 period in the other hand. Since the Phenoclim dataset concerns mountainous regions only and started in 2005, in order to avoid a bias due to altitude differences between the different time periods, only data inferior to 600 m a.s.l were kept from this source of data for the analysis. Means and standard deviations of these differences are presented in Fig. 2.

## Supplementary Information

Below is the link to the electronic supplementary material.

Supplementary Material 1

Supplementary Material 2

Supplementary Material 3

Supplementary Material 4

Supplementary Material 5

Supplementary Material 6The link to this file is missing

## Data Availability

Request of the data should be addressed to Isabelle Chuine [isabelle.chuine@cefe.cnrs.fr](mailto: isabelle.chuine@cefe.cnrs.fr) . Data used for this study are available in Tables S1, S2, S3. References of historical documents used I this study are provided in Table 2 and References. All data are also available under DOI 10.57745/QD1OR6 in repository [https://entrepot.recherche.data.gouv.fr/dataset.xhtml?persistentId=doi:10.57745/QD1OR6].
