# Supplementary material for: Living things are showing increasing anomalies in their seasonal activity, which could disrupt the dynamics of biodiversity and ecosystems
Source: Sci Rep. 2025 Sep 25;15:32860. doi: 10.1038/s41598-025-16585-2 (PMC12464327; doi:10.1038/s41598-025-16585-2)
Supplement: Supplementary file 5 — Supplementary Material 5 [file 41598_2025_16585_MOESM5_ESM.docx]

**Title:** Living things are showing increasing anomalies in their seasonal activity, which could disrupt the dynamics of biodiversity and ecosystems

**Authors :** Isabelle Chuine^1^, Iñaki Garcia de Cortazar-Atauri^2^, Frédéric Jean^3^, Colin Van Reeth^4^

**Authors affiliation :**

^1^CEFE, Univ Montpellier, CNRS, EPHE, IRD, Montpellier, France

^2^INRAE, US AgroClim, F-84914, Avignon, France

^3^INRAE, URFM, F-84914, Avignon, France

^4^CREA Mont Blanc, Chamonix, France

**Corresponding Author:** Isabelle Chuine [isabelle.chuine@cefe.cnrs.fr](mailto:isabelle.chuine@cefe.cnrs.fr)

**Supplementary Information**

Table S1. S1a. Reports of abnormal phenological events since 2015 in traditional media and social media worldwide. Keywords used: in English: strange flowering/blooming, early flowering, in French: floraison précoce/atypique, in Spanish: floracion precoz/atipica. S1b. Sources associated to each report in Table S1a.

Table S2. Reports of abnormal phenological event since 2015 in France from citizen science programs.

Table S3. Phenological data reported since 1901 analyzed in Fig. 2. Data are from the citizen science programs Observatoire des Saisons (https:// obs-saisons.fr), Phenoclim (https://phenoclim.org/), Abiome (https://abiome.assoconnect.com/page/1005353-accueil), Orchisauvage (https://www.orchisauvage.fr/) and from the TEMPO portal (https://tempo.pheno.fr).

Figure S1. Temperature anomaly (relative to 1961-1990) of September-October (grey) and November-February (black) from 1960 to 2020. For each year *n* are represented the mean temperature of September-October (year *n*-1) and the mean temperature of November-December (year n-1)-January-February (year *n)*.
