# Supplementary figures and images for: Living things are showing increasing anomalies in their seasonal activity, which could disrupt the dynamics of biodiversity and ecosystems
Source: Sci Rep. 2025 Sep 25;15:32860. doi: 10.1038/s41598-025-16585-2 (PMC12464327; doi:10.1038/s41598-025-16585-2)

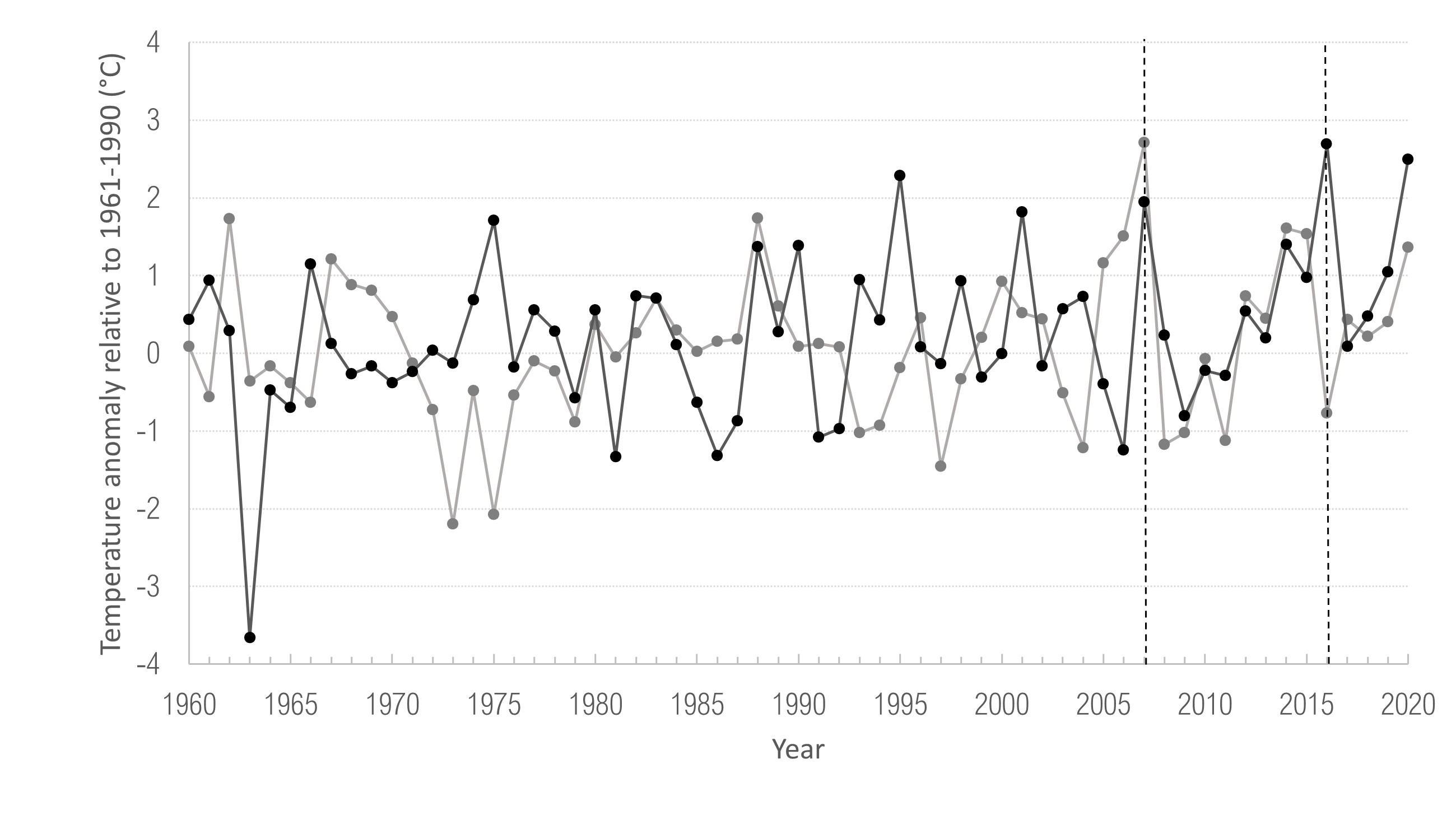

Supplement: Supplementary file 6 — Supplementary Material 6The link to this file is missing [file 41598_2025_16585_MOESM6_ESM.jpg]
